# Enhancing Bone–Cartilage Interface Healing in Osteochondral Autograft Transplantation: Effects of BMAC Augmentation and Rehabilitation Protocols

**DOI:** 10.3390/life15071066

**Published:** 2025-07-03

**Authors:** Robert Gherghel, Ilie Onu, Ana Onu, Ioana-Irina Rezus, Ovidiu Alexa, Daniel Andrei Iordan, Luana Andreea Macovei, Elena Rezus

**Affiliations:** 1 Department of Orthopedics and Trauma Surgery, Piatra Neamt Emergency Hospital, 700115 Piatra Neamt, Romania; gherghelrobert@yahoo.com; 2 Doctoral School, University of Medicine and Pharmacy “Grigore T. Popa”, 700454 Iasi, Romania; chirila.ana@d.umfiasi.ro; 3 Departments of Orthopedics and Physiotherapy, Medlife-Micromedica Clinic, 610119 Piatra Neamt, Romania; 4 Department of Biomedical Sciences, Faculty of Medical Bioengineering, University of Medicine and Pharmacy “Grigore T. Popa”, 700454 Iasi, Romania; 5 Department of Radiology, University of Medicine and Pharmacy, Iasi “Grigore T. Popa”, 700115 Iasi, Romania; ioanairinarezus@yahoo.co.uk; 6 Department of Orthopaedic and Traumatology, University of Medicine and Pharmacy, Iasi “Grigore T. Popa”, 700115 Iasi, Romania; ovidiu.alexa@umfiasi.ro; 7 Center of Physical Therapy and Rehabilitation, “Dunărea de Jos” University of Galati, 800008 Galati, Romania; daniel.iordan@ugal.ro; 8 Department of Individual Sports and Kinetotherapy, Faculty of Physical Education and Sport, “Dunarea de Jos” University of Galati, 800008 Galati, Romania; 9 Department of Rheumatology and Rehabilitation, University of Medicine and Pharmacy “Grigore T. Popa”, 700115 Iasi, Romania; elena.rezus@umfiasi.ro; 10 Ist Rheumatology Clinic, Clinical Rehabilitation Hospital, 14 Pantelimon Halipa Street, 700661 Iasi, Romania

**Keywords:** osteochondral defect, mosaicplasty, osteochondral autologous transplantation (OAT), bone marrow aspirate concentrate (BMAC), cartilage repair, rehabilitation, MOCART 2.0 score, MRI evaluation

## Abstract

This study aimed to evaluate the effectiveness of different rehabilitation protocols following osteochondral autograft transplantation (OAT) in patients with focal osteochondral defects of the femoral condyle, using the MOCART 2.0 knee score as a primary imaging outcome. Twenty-nine patients were divided into three groups: Group 1 (*n* = 9) received OAT with bone marrow aspirate concentrate (BMAC) and a 12-week two-phase rehabilitation program; Group 2 (n = 11) received OAT with a 12-week program without BMAC; and Group 3 (n = 9) received OAT with a shortened 6-week program. At the 12-month follow-up, Group 1 demonstrated a superior cartilage repair quality, with the highest mean MOCART 2.0 score (96.1), compared to Group 2 (80.2) and Group 3 (71.7). Notably, complete defect filling was observed in five patients in Group 1 versus four in Group 2 and only one in Group 3. The integration and surface integrity were also better preserved in Group 1. The addition of BMAC and an extended, progressive rehabilitation protocol significantly enhanced the morphological cartilage repair parameters. These results suggest that a biologically enhanced and prolonged recovery plan may offer a greater structural restoration of cartilage after OAT than conventional or accelerated protocols.

## 1. Introduction

Large, deep cartilage defects in load-bearing joints such as the knee and ankle represent a significant clinical challenge due to the limited intrinsic healing capacity of hyaline cartilage. Left untreated, these lesions can lead to progressive joint degeneration, pain, functional impairment, and ultimately, osteoarthritis [1,2]. To address this issue, osteochondral autograft transplantation (OAT)—also widely known as mosaicplasty—has emerged as a well-established surgical technique aimed at restoring the joint surface integrity and delaying or preventing joint deterioration.

Originally described by Hangody et al. [1,2], the procedure involves harvesting one or more cylindrical osteochondral plugs from non-weight-bearing areas of the femoral condyle and implanting them into corresponding sockets prepared at the defect site. This technique offers a biological and mechanical advantage by transferring autologous tissue that includes both viable subchondral bone and hyaline cartilage, ensuring structural and functional integration at the recipient site [3,4].

The cylindrical geometry of the plugs allows for precise anatomical congruency, which is critical for restoring the joint surface integrity and achieving immediate mechanical stability after implantation [4,5]. This stability is enhanced by the press-fit fixation technique, which relies on tightly inserting the plugs into predrilled sockets without the need for additional fixation devices. As a result, the transplanted graft can endure physiological load transmission immediately postoperatively, facilitating early mobilization and weight-bearing [6,7].

Histological studies have confirmed that the bone portion of the graft achieves complete osseous integration with the host tissue [8], while the cartilage portion maintains viable chondrocytes essential for long-term function and resistance to degeneration [9]. Moreover, when the press-fit technique is performed correctly and combined with low-velocity, multiple-impact insertion methods, it minimizes chondrocyte death and preserves the native architecture of the cartilage. This ensures that the transplanted plugs remain histologically organized and free from hypertrophy or degenerative changes [8,9,10].

Unlike cartilage regeneration techniques, OAT provides immediate resurfacing with native hyaline cartilage [3]. Hyaline cartilage is mechanically superior to fibrocartilage produced by microfracture or abrasion arthroplasty [11]. Therefore, the donor plug restores the articular surface with optimal biomechanical properties [4]. The curvature and thickness of the graft match that of the native cartilage, reducing joint incongruity [12]. A mismatch of less than 1 mm between the graft and surrounding cartilage correlates with improved integration [13]. The tight fit prevents lateral micromotion, a factor that might impair osseous incorporation [8]. Vertical stability is ensured by the congruent cylindrical geometry, limiting graft displacement [6]. Press-fit insertion minimizes extrusion and promotes stable healing [9]. Clinical studies show the long-term survival of grafts without signs of loosening or degeneration. Emre et al. (2018) reported no signs of graft failure or pain 18 months postoperatively [14].

Return-to-sport rates in athletes are high after OAT, with up to 92% resuming previous performance levels [3]. Functional scores post-surgery remain stable or improve, supporting the functional longevity of the graft [6]. Pain-free joint function persists in the majority of patients for years after implantation. With proper surgical technique and patient selection, complications such as graft failure or donor site morbidity (DSM) are minimal [12]. The donor plug restores both the structural and mechanical properties of the osteochondral unit [11]. Unlike regenerative procedures, OAT does not depend on time-consuming biological remodeling [3]. This results in a faster return to normal activities and sports participation. The preserved chondrocyte viability ensures a sustained cartilage function and resistance to degeneration [7]. Studies confirm that plugs survive functionally and biologically for over 10 years in many cases [6]. These features make the donor plug an effective and durable solution for focal osteochondral defects [2].

A retrospective single-center study evaluated the long-term outcomes of knee OAT in 55 soccer players treated between 1992 and 2011. The average follow-up period was over 17 years, with assessments including the International Knee Documentation Committee (IKDC), Bandi, Tegner, and Magnetic Resonance Observation of Cartilage Repair Tissue (MOCART) scores. The results showed sustained functional outcomes, with an average IKDC of 68.5 and MOCART of 70.8, and with 80% of patients reporting good Bandi scores. The mean defect size was 2.08 cm^2^ and the average return to sport was approximately 8 months. Overall, OAT using mosaicplasty demonstrated durable benefits in athletic populations, making it a viable option for treating small-to-medium knee lesions in soccer players [15].

While the procedure offers the advantage of restoring hyaline-type cartilage and addressing subchondral defects, it requires sectioning intact articular cartilage at a second site, which raises issues of DSM [16]. DSM remains a significant concern in OAT despite the procedure’s success in restoring joint function and alleviating pain [17]. DSM encompasses complications such as pain, functional limitations, and osteoarthritis (OA) at the graft harvest site, with reported incidence rates ranging from 6% to 20%. This wide variability suggests a complex interplay of contributing factors that are not yet fully understood. The current literature presents conflicting evidence regarding the role of patient-specific variables in predicting the DSM risk [18,19,20].

A recent study assessed the effectiveness of combining OAT with BMAC and varying rehabilitation protocols. Thirty-seven patients with femoral condyle chondral or osteochondral lesions >3 cm^2^ were randomized into three groups. Group 1 received mosaicplasty with BMAC and a 12-week rehab program, Group 2 received mosaicplasty only with a 12-week rehab program, and Group 3 had mosaicplasty with a shorter 6-week rehab program. Significant improvements in the Western Ontario and McMaster Universities Arthritis Index (WOMAC) and Visual Analogue Scale (VAS) scores were observed in Group 1 compared to the other groups, particularly at intermediate and final follow-ups. The knee range of motion (ROM) also improved significantly in Group 1 versus Groups 2 and 3, especially after the intermediate and final assessments. Overall, combining BMAC augmentation with an extended rehabilitation protocol led to superior outcomes in pain, function and joint mobility for patients with knee chondral defects [21].

The objective of this study was to evaluate the effectiveness of different rehabilitation protocols following OAT in patients with focal osteochondral defects of the femoral condyle, with graft integration assessed using the MOCART 2.0 knee score. Building upon our previous work [21], this manuscript specifically focuses on 12-month imaging outcomes to assess the quality of graft integration using the MOCART 2.0 system.

We hypothesized that patients undergoing OAT augmented with BMAC and a structured 12-week, two-phase rehabilitation program would demonstrate complete and stable osteochondral graft integration without subchondral bone loss, as evidenced by MOCART 2.0 scores. In contrast, patients receiving the same rehabilitation protocol without BMAC augmentation may show satisfactory graft integration on imaging, but are expected to experience residual clinical symptoms such as joint instability or mechanical cracking. Furthermore, patients following a shortened 6-week rehabilitation program are anticipated to have a higher risk of incomplete graft integration or partial failure, with correspondingly lower MOCART scores.

## 2. Materials and Methods

### 2.1. Study Design and Procedure Steps

This single-center retrospective study was conducted from January 2021 to December 2024 in the orthopedics and physiotherapy departments of Micromedica Clinic and Piatra Neamt County Emergency Hospital, Orthopedics. This study was approved by the Scientific Research Ethics Committee of Clinics Micromedica (Approval no. 24 dated 14 January 2022) and was conducted by the principles of the Declaration of Helsinki. All patients signed informed consent for participation.

Twenty-nine patients with a moderate level of physical activity, diagnosed with symptomatic chondral or osteochondral defects ≤ 3 cm in size, localized in the weight-bearing area of the femoral condyle, were initially selected. Imaging MRI investigations confirmed the diagnosis.

Inclusion criteria were age less than 50 years, normal values of inflammatory markers, absence of intra-articular infiltrations with corticosteroids or viscoelastic products in the last 12 months, and absence of recent episodes of synovitis.

Exclusion criteria included age > 50 years, cartilage defects > 3 cm, inability to harvest graft from non-weight-bearing areas, presence of advanced osteoarthritis, osteoporosis, obesity, metabolic or rheumatic diseases, ligamentous injuries, limited flexion ROM (<120°), or moderate hydrarthrosis.

### 2.2. Participants

Twenty-nine participants were allocated into three groups as follows (Table 1):

Group 1 (*n* = 9): OAT associated with a two-phase (12 weeks) rehabilitation program and augmentation with BMAC;

Group 2 (*n* = 11): OAT and two-stage rehabilitation (12 weeks) without biological augmentation;

Group 3 (*n* = 9): OAT and single-phase rehabilitation program (6 weeks).

The rehabilitation program was structured in two phases: in the first phase (0–6 weeks), patients underwent non-load-bearing physiotherapy exercises, electrotherapy, and Deep Oscillation. In the second phase (6–12 weeks), active exercises with assisted partial loading, muscle electrostimulation, and quadriceps toning exercises were introduced [16].

All patients were monitored for 12 months postoperatively. The final evaluation was conducted in 2024 and included a clinical examination and MRI evaluation.

### 2.3. Surgical Treatment and BMAC Augmentation

The surgical procedures were conducted at the Piatra Neamț Emergency County Hospital. Pre- and postoperative radio-imaging investigations were performed at the Micromedica Clinic, utilizing 1.5 Tesla MRI systems.

Mosaicplasty was performed using the Arthrex OATS^®^ disposable kit (Arthrex GmbH, Munich, Germany), while BMAC augmentation was carried out using the BioCUE^®^ BMAC kit (Zimmer Biomet, Warsaw, IN, USA).

Following the initial arthroscopic examination and debridement, the size and location of the chondral defect were assessed. Osteochondral grafts were harvested from the peripheral non-weight-bearing area of the trochlea. For graft insertion, a drill guide was placed perpendicular to the joint surface, allowing for the precise reaming of the recipient socket. Grafts were spaced ~3 mm apart to prevent tunnel confluence and condylar loosening. Proper depth and orientation were ensured to replicate the native joint curvature.

In Group 1, BMAC augmentation followed OAT. Bone marrow was aspirated from the Anterior Superior Iliac Spine (ASIS) with the patient in a supine position and the knee flexed to 90°, supported on a surgical platform. A single needle insertion with 2–3 redirections was used, yielding ~15–20 mL of marrow per pass, collected into a 60 mL syringe. The aspirate underwent centrifugation at 2400 rpm for 10 min (to remove platelet-poor plasma), followed by a second spin at 3400 rpm for 6 min. The final BMAC layer was injected intra-articularly under ultrasound guidance.

Rehabilitation was initiated 72 h postoperatively, following a 6- or 12-week protocol, depending on group allocation.

### 2.4. Postoperative Rehabilitation

Phase 1 (weeks 0–6) focused on pain relief, restoring passive knee flexion–extension, and regaining quadriceps control. Pain and inflammation were managed using 40 min of conventional transcutaneous electrical nerve stimulation (TENS) (100 Hz, 100 μs), 8 min of ultrasound (US) at 0.2–0.3 W/cm^2^ (1 MHz, 10% duty cycle), 15 min of Deep Oscillation (10 min at 170–250 Hz, 5 min at 70–90 Hz), and 15–20 min of cryotherapy at 4 °C. Patients used crutches and orthoses (0–30°), with passive mobilization via CPM or physiotherapist-assisted exercises during the first 2 weeks. From week 3, stationary cycling without resistance and isometric quadriceps exercises were introduced. Weight-bearing was prohibited [21].

Phase 2 (weeks 6–12) aimed to improve muscle strength and endurance. Orthoses were removed and progressive weight-bearing was allowed within pain limits. Electrotherapy included 15 min of low-frequency Electrical Muscle Stimulation (EMS) followed by 15 min of medium-frequency Kotz stimulation with isometric contractions. Strengthening exercises included ankle-weight training, partial squats, and wall slides (0–30°), along with full-ROM cycling. High-impact, pivoting, and full-extension loading exercises remained contraindicated [21].

### 2.5. Imaging Assessment and Use of the MOCART 2.0 Score

The MOCART 2.0 score was used to qualitatively and quantitatively assess the articular cartilage regeneration in patients treated with OAT. This score enables a standardized analysis of the repaired cartilage tissue by examining multiple morphological and structural criteria, including surface integrity, degree of filling, signal of the repaired cartilage, and subchondral bone status [22,23].

The score includes morphologic and structural parameters, each with a predetermined numerical value, with a maximum score of 100 points [22,23]:

- Complete or minor hypertrophy (100–150% fill): 20 pts; major hypertrophy ≥150% or 75–99% fill: 15 pts; 50–74%: 10 pts; 25–49%: 5 pts; and <25% or delamination: 0 pts.
- Complete integration: 15 pts; split-like defect ≤2 mm: 10 pts; defect >2 mm but <50%: 5 pts; and ≥50%: 0 pts.
- Surface intact: 10 pts; irregular <50%: 5 pts; and irregular ≥50%: 0 pts.
- Structure homogeneous: 10 pts; inhomogeneous: 0 pts.
- Signal normal: 15 pts; minor abnormal: 10 pts; and severe abnormal: 0 pts.
- No bony defect/overgrowth: 10 pts; minor (<thickness or <50%): 5 pts; and major (≥thickness or ≥50%): 0 pts.
- No subchondral change: 20 pts; minor edema <50%: 15 pts; severe edema ≥50%: 10 pts; and cyst ≥5 mm or osteonecrosis: 0 pts.

To allow a correct application of the MOCART 2.0 score, the MRI imaging protocols were adapted accordingly. Investigations were performed on a Siemens Magnetom Aera 1.5 T, using a dedicated 16-channel knee coil. The following sequences were included: proton-density-weighted turbo spin-echo (PDw TSE) without and with fat suppression (FS), and T1-weighted Turbo Spin Echo (T1w TSE), respectively, with technical parameters modified as follows:

- TE: 25–35 ms for PDw sequences, 10–15 ms for T1w.
- TR: 3000–4000 ms for PDw, 500–800 ms for T1w.
- Section thickness: 1.5–3 mm, with minimum intersectional spacing (gap 0–10%).
- Slices: 25–48.
- FOV: 140–160 mm, with a minimum acquisition matrix of 384 × 384.
- Orientation: sagittal, supplemented by transverse sequences.

Assessments were performed at 12 months postoperatively by two independent radiologists specializing in musculoskeletal imaging to ensure the reproducibility and objectivity of the MOCART 2.0 scoring.

### 2.6. Statistical Analysis

Statistical analysis was performed using IBM SPSS Statistics, version 25.0 (IBM Corp., Armonk, NY, USA). The normality of continuous variables was assessed using the Shapiro–Wilk test. Data were expressed as means and standard deviations for normally distributed variables and as medians and interquartile ranges for non-parametric data.

To evaluate differences in the MOCART 2.0 subscores and total score between the three treatment groups, a one-way analysis of variance (ANOVA) was applied. For parameters that did not meet the assumptions of normal distribution, the Kruskal–Wallis test was used as a non-parametric alternative.

When significant group differences were identified, post hoc comparisons were performed using the Tukey HSD test for parametric data and the Dunn–Bonferroni correction for non-parametric data.

For the analysis of graft survival, defined categorically as achieving a total MOCART 2.0 score ≥ 90 points, Fisher’s exact test was used to compare proportions between groups due to the small sample sizes. All statistical tests were two-tailed and a *p*-value < 0.05 was considered statistically significant.

## 3. Results

At the 12-month follow-up, the quality of the cartilage repair tissue was evaluated using the MOCART 2.0 scoring system, adapted to assess outcomes specific to OAT procedures.

**Figure 1:**
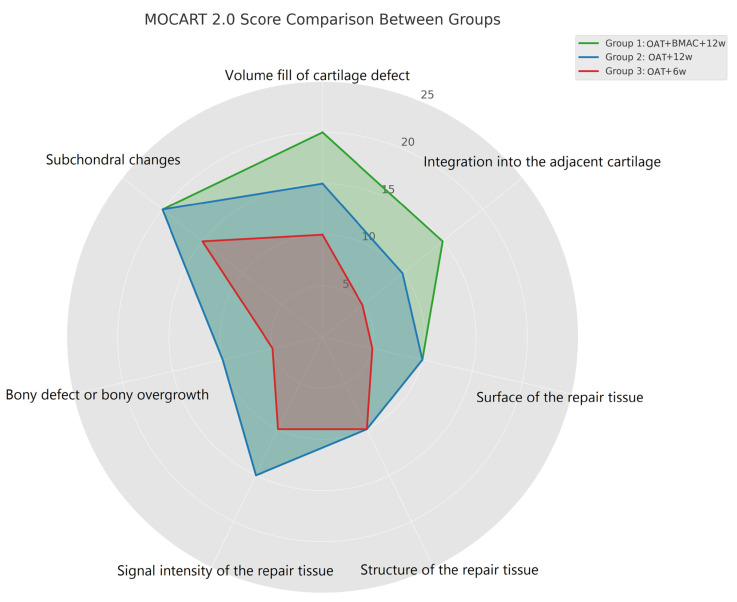
Radar chart of MOCART 2.0 subscores for the three treatment groups. Group 1 (OAT + BMAC + 12w) showed the highest overall scores, followed by Group 2 (OAT + 12w) and Group 3 (OAT + 6w), highlighting the positive impact of BMAC and extended rehabilitation duration on cartilage repair quality.

A clear difference emerged between the three groups under investigation, as shown in Table 2 and visualized in the radar chart (Figure 1). Group 1, which underwent OAT combined with BMAC and followed a structured 12-week rehabilitation protocol, demonstrated significantly superior outcomes across nearly all MOCART parameters. The mean total MOCART score for this group was 96.1, reflecting the nearly complete repair and morphological restoration of the defect. In comparison, Group 2 (OAT + 12-week rehabilitation without BMAC) had a lower mean score of 80.2, while Group 3 (OAT with only a 6-week rehabilitation program) recorded the lowest value, 71.7, indicating suboptimal integration and repair tissue characteristics.

In detail, the defect fill volume was complete or presented minor hypertrophy in over half the patients in Group 1 (55.5%), compared to 36.3% in Group 2 and only 11.1% in Group 3. Integration with adjacent cartilage was notably superior in Group 1, with complete integration achieved in 100% of cases, while this was only observed in 45.4% of Group 2 and 22.2% of Group 3 patients. The surface integrity of the repair tissue followed a similar trend: an intact surface was present in 66.6% of Group 1, whereas major irregularities (defined as affecting ≥50% of the repair zone) were recorded in multiple cases in Group 3. The structure of the repair tissue was homogeneous across all patients in Groups 1 and 2 and in 66.6% of Group 3, suggesting that while the structure per se might not be significantly affected by the intervention, its surface morphology and integration are considerably improved when BMAC and extended rehabilitation are included.

Regarding the MRI signal intensity, a parameter reflecting the tissue maturation and composition, a normal signal was present in 77.7% of both Group 1 and Group 2, while Group 3 exhibited a lower percentage (55.5%), including one case with a severe fluid-like signal, which is typically associated with immature or non-hyaline-like repair tissue. Subchondral bone defects or protrusions were absent in most patients in Groups 1 and 2, but three patients from Group 3 showed residual or emerging bone alterations. Furthermore, subchondral edema-like changes, which reflect inflammation or incomplete bone remodeling, were least frequent in Group 1 (11.2%) and most frequent in Group 3 (55.6%), where two patients had severe changes.

The statistical analysis confirmed these differences: one-way ANOVA revealed statistically significant differences across groups for the defect fill (*p* < 0.01), integration with adjacent cartilage (*p* < 0.01), surface integrity (*p* = 0.03), and subchondral signal changes (*p* = 0.04). Interestingly, no statistically significant difference was found regarding the structure of the repair tissue (F = 0.0, *p* = 1.00), reinforcing the idea that BMAC and the rehabilitation duration primarily affect the integration, surface, and subchondral remodeling rather than intrinsic tissue homogeneity. A trend toward significance was also observed in the MRI signal intensity (F ≈ 3.2, *p* ≈ 0.07), suggesting a possible favorable maturation pattern in the BMAC group, which might reach statistical significance in studies with larger sample sizes.

These findings are concisely illustrated in Figure 1, where the radar chart visually compares the performance of each group across the seven MOCART 2.0 parameters. The green polygon representing Group 1 consistently extends further along the axes corresponding to the defect fill, cartilage integration, subchondral remodeling, and signal intensity, demonstrating superior regenerative outcomes. This graphical visualization supports the conclusion that the addition of BMAC to OAT, in conjunction with an extended and structured 12-week rehabilitation program, leads to the significantly improved morphological and functional integration of the graft, suggesting that both biological and mechanical optimization strategies are essential to enhancing cartilage repair in clinical practice.

### Graft Survival Analysis

To evaluate the long-term integration and viability of osteochondral plugs, graft survival was defined as a total MOCART 2.0 score equal to or greater than 90 points at the 12-month follow-up. This threshold reflected optimal defect filling, complete integration into adjacent cartilage, an intact repair tissue surface, normal MRI signal intensity, and absence of bony or subchondral changes.

Survival rates varied significantly between groups. In Group 1 (OAT + BMAC + 12-week rehab), seven out of nine patients (77.8%) met the criteria for successful graft survival. In contrast, only 3 out of 11 patients (27.3%) in Group 2 (OAT + 12-week rehab) and 1 out of 9 patients (11.1%) in Group 3 (OAT + 6-week rehab) achieved similar outcomes (Figure 1).

Statistical analysis using Fisher’s Exact Test revealed a significant difference in graft survival rates between groups (*p* ≈ 0.01), indicating that both biological augmentation with BMAC and an extended two-phase rehabilitation protocol substantially increased the likelihood of durable osteochondral graft incorporation.

These findings emphasize the importance of combining regenerative biologics with structured, prolonged rehabilitation to maximize the structural and functional integration of osteochondral plugs, ultimately reducing the risk of the donor site morbidity and graft failure.

To better characterize the long-term integration of osteochondral plugs, graft survival was defined as achieving a total MOCART 2.0 score ≥ 90 points. This threshold reflects nearly ideal cartilage regeneration, including complete fill, stable integration, a smooth surface, and absence of subchondral pathology.

In Group 1, seven out of nine patients (77.8%) met the survival criteria. In Group 2, only 3 out of 11 patients (27.3%) reached the same threshold. In Group 3, just one of nine patients (11.1%) demonstrated sufficient graft maturity.

Fisher’s exact test indicated a statistically significant difference in survival rates between groups (*p* ≈ 0.01), confirming that both biological and mechanical factors contribute substantially to graft preservation and successful integration.

## 4. Discussion

This study evaluated the outcomes of OAT across three therapeutic protocols, focusing on graft integrity as measured by the MOCART 2.0 score at 12 months. Group 1, which received BMAC and a structured 12-week rehabilitation program, achieved the highest MOCART scores (mean 96.1), indicating near-complete defect restoration. The addition of BMAC, rich in mesenchymal stem cells and growth factors, likely enhanced donor plug survival by promoting chondrogenesis, matrix deposition, and subchondral bone repair.

Rehabilitation played a crucial role in graft integration. Group 2, which followed the same rehab protocol without BMAC, showed lower but acceptable outcomes (mean score 80.2), emphasizing that progressive mechanical loading supports graft maturation. In contrast, Group 3, with only a 6-week rehab program and no BMAC, had significantly poorer results (mean score 71.7), suggesting an inadequate time for full osteochondral integration. These findings highlight the synergistic benefits of biological augmentation and structured rehabilitation in enhancing long-term viability and the integration of the donor plug.

Despite risks, OAT remains advantageous compared to microfracture as it promotes hyaline cartilage repair rather than fibrocartilage, which is mechanically inferior and more prone to degeneration. Long-term studies show success rates of up to 72% at 10 years, particularly in young, active patients, when the procedure is properly indicated and rehabilitation protocols are followed [24,25,26].

Recent evidence suggests that biological augmentation with BMAC may enhance regeneration at both graft and donor sites. In the current study, the group receiving OAT combined with BMAC and a 12-week rehabilitation protocol experienced fewer complications related to DSM, such as joint instability and mechanical symptoms [21]. The regenerative potential of BMAC, rich in mesenchymal stem cells, cytokines, and growth factors, likely contributed to improved integration and healing [27,28].

BMAC has emerged as a promising biologic therapy for symptomatic knee OA. Clinical studies have shown that BMAC injections significantly improve pain and functional outcomes, often outperforming alternatives like platelet-rich plasma (PRP) and microfragmented adipose tissue [29,30].

Skowroński et al. demonstrated that a one-stage reconstruction combining BMAC with collagen membranes led to marked functional improvements in 52 out of 54 patients, with an average gain of 25 points in KOOS and 35 points in Lysholm scores at one year. No complications were reported and BMAC was considered more cost-effective than autologous chondrocyte implantation [31].

Krych et al. found that scaffold implantation augmented with BMAC resulted in superior cartilage maturation compared to PRP or scaffold-only groups. MRI imaging at 12 months showed better cartilage fill and T2 values closer to native hyaline cartilage, indicating enhanced early repair [32].

Conversely, Wang et al. concluded that BMAC did not significantly improve osseous integration in osteochondral allograft transplantation, suggesting that its role in graft–host bone healing remains uncertain and warrants further study [33].

Solheim et al. reported similar long-term outcomes for OAT procedures in both patellofemoral and femoral condyle regions, with no significant survival differences between locations. Despite a 50% failure rate at 18 years, many patients maintained functional joints, supporting the durability of OAT [27].

In a separate clinical study, Zhou et al. evaluated 60 patients undergoing knee arthroscopy with mesenchymal stromal cell concentrates. Significant improvements in the VAS and WOMAC scores were observed up to 12 months postoperatively, indicating short-term pain relief and functional gains in patients with chondral lesions. These clinical improvements were corroborated by significantly higher MOCART scores in the experimental group at 12 months. The alignment between subjective clinical outcomes and objective MRI-based assessments supports the hypothesis that biological augmentation enhances cartilage repair. Elevated MOCART scores reflect more complete defect filling, improved surface congruity, and a better overall cartilage quality. Unlike functional scores alone, the MOCART system offers morphological evidence of graft maturation and structural integration. The absence of significant differences in the WOMAC stiffness scores suggests that certain joint properties may require longer-term follow-up or alternative evaluation metrics. Overall, the integration of imaging data strengthens the clinical interpretation of outcomes and underscores the utility of an advanced MRI in tracking cartilage regeneration [34].

TENS therapy is commonly found postoperatively in orthopedic surgery for pain relief. TENS involves rectangular single-phase or biphasic currents that stimulate large-diameter A-β afferents, thereby inhibiting nociceptive activity at the spinal level [35,36]. Zhou et al. reported that electrical stimulation can enhance extracellular matrix production, cell proliferation, and chondrogenic differentiation in vitro, suggesting a role in cartilage repair via tissue engineering [37]. Moreover, TENS has been shown to reduce pro-inflammatory cytokines post-treatment, highlighting additional mechanisms of analgesia [38].

Deep Oscillation^®^ (PHYSIOMED, Schnaittach, Germany) has demonstrated significant effects in musculoskeletal rehabilitation, including pain reduction, anti-inflammatory activity, edema reabsorption, and improved lymphatic drainage [39,40]. Vladeva et al. confirmed its effectiveness in improving ROM and decreasing pain and swelling in early post-arthroplasty rehabilitation [41].

US therapy, widely used in musculoskeletal physiotherapy, provides non-invasive tissue penetration with benefits such as collagen stimulation, inflammation modulation, and cartilage repair [21,42,43,44]. Low-intensity pulsed US (0.2 W/cm^2^, 10% duty cycle) has shown bio-stimulatory effects on fibroblasts and modulated IL-6 expression, supporting its therapeutic impact on tissue healing [45].

Cryotherapy is a safe, cost-effective postoperative intervention for managing inflammation, edema, and pain [46]. van Ooij et al. demonstrated its role in enhancing early knee flexion and accelerating rehabilitation post-TKA [47]. In this study, cryotherapy was implemented post-therapy to reduce the exercise-induced joint temperature and to protect graft sites by limiting edema and pain [21].

Physiotherapy exercises aim to preserve the joint function and restore the lower limb strength, stability, and coordination. For effective chronic inflammation control, guidelines recommend ≥150 min/week of moderate or ≥75 min/week of vigorous-intensity exercise [48,49]. The Massachusetts General Hospital rehabilitation protocol includes preoperative stationary biking, followed by postoperative continuous passive motion (CPM) (0–50°, progressing to 100°), quadriceps setting, heel slides, and ankle pumps [50]. Exercise has important anti-inflammatory and antioxidant effects. Regular physical activity reduces levels of proinflammatory cytokines such as IL-6 and TNF-α and stimulates the production of endogenous antioxidant enzymes (e.g., superoxide dismutase, catalase, and glutathione peroxidase). Through these mechanisms, exercise contributes to decreasing oxidative stress, improving metabolic function and reducing the risk of chronic diseases [51].

Several limitations must be acknowledged. First, the relatively small sample size, divided into three groups, limits the statistical power and generalizability of the findings. Additionally, the monocentric nature of this study may affect the uniformity of the treatment and evaluation protocols.

Another notable limitation is the absence of clinical and functional outcome scores that would capture the patient’s perception of pain and the joint function. In a previous study we conducted, we used scores such as the VAS, WOMAC, ROM, and MMT, which provided valuable insights into subjective and functional progression [21]. Incorporating such measures in the present study would have allowed for a more robust correlation between the imaging outcomes (as assessed by MOCART 2.0) and clinical status, offering a more holistic perspective on the treatment efficacy.

Moreover, the lack of a histological assessment of the cartilage grafts and the absence of a postoperative arthroscopic evaluation limit the direct confirmation of the graft integration and tissue quality. A long-term functional follow-up (beyond 12 months) was also not conducted, which could have provided insights into the durability of the observed benefits.

Despite these limitations, our study offers meaningful contributions by comparing three distinct postoperative rehabilitation protocols after OAT, highlighting the potential of combining cellular therapy with progressive physiotherapy to optimize cartilage regeneration.

## 5. Conclusions

The results of this study indicate that combining osteochondral OAT with BMAC and a structured 12-week two-phase rehabilitation program significantly improves cartilage repair, as evidenced by higher MOCART 2.0 scores. Patients in Group 1 (OAT + BMAC + 12-week rehab) achieved better outcomes in the defect filling, tissue integration, surface integrity, and subchondral bone health compared to those who received OAT alone with either standard (12-week) or accelerated (6-week) rehabilitation. The superior mean MOCART score in Group 1 (96.1 vs. 80.2 and 71.7) supports the hypothesis that biologically enhanced repair combined with a prolonged, progressive recovery plan promotes superior structural regeneration.

These findings underscore the crucial role of both biological augmentation and sufficient rehabilitation duration in achieving optimal postoperative outcomes following cartilage repair procedures. Future research with larger cohorts and long-term follow-up is warranted to confirm the durability and functional relevance of these imaging findings.

Conversely, abbreviated rehabilitation protocols were associated with poorer graft incorporation, residual subchondral abnormalities, and incomplete tissue maturation. These results underscore the importance of a multimodal approach to cartilage repair, combining biological augmentation with tailored physiotherapeutic strategies to maximize structural and functional outcomes.

Future prospective studies with larger cohorts, longer follow-up periods, and integrated clinical outcome measures are warranted to validate these findings and develop standardized protocols for managing osteochondral defects.

## Figures and Tables

**Table 1:** Group allocation and rehabilitation protocols.

Group	Treatment	Rehabilitation Protocol	Patients (*n*)
Group 1	OAT with BMAC augmentation	Two-phase, 12 weeks: Phase I (0–6 weeks): non-load-bearing physiotherapy exercises, TENS, ultrasound, Deep Oscillation Phase II (6–12 weeks): partial weight-bearing, electrostimulation, quadriceps toning	9
Group 2	OAT without BMAC	Two-phase, 12 weeks: same protocol as Group 1 without biological augmentation	11
Group 3	OAT without BMAC	Single-phase (Phase I), 6 weeks: non-load-bearing physiotherapy exercises, TENS, ultrasound, Deep Oscillation, without progression to loading or electrostimulation	9

**Table 2:** Magnetic resonance observation of cartilage repair tissue (MOCART) 2.0 knee score.

MOCART 2.0: Cartilage Repair Tissue Assessment (Knee)		Points	Group 1 OAT + BMAC + 12w (*n =* 9)	Group 2 OAT + 12w (*n* = 11)	Group 3 OAT + 6w (*n =* 9)
Volume fill of cartilage defect	Complete filling or minor hypertrophy	20	5	4	1
	Major hypertrophy (≥150%) or 75% to 99% filling	15	2	4	3
	50% to 74% filling	10	2	2	3
	25% to 49% filling	5	0	1	2
	<25% filling or complete delamination	0	0	0	0
Integration into the adjacent cartilage	Complete 15p	15	5	5	2
	Split-like defect ≤2 mm	10	3	5	3
	Defect >2 mm but <50% of repair tissue length	5	1	1	4
	Defect ≥50% of repair tissue length	0	0	0	0
Surface of the repair tissue	Intact	10	6	6	3
	Damaged: <50% of the repair tissue diameter	5	3	4	3
	Damaged: ≥50% of the repair tissue diameter	0	0	1	3
Structure of the repair tissue	Homogeneous 10p		9	9	6
	Inhomogeneous 0p		0	2	3
Signal intensity of the repair tissue	Normal	15	7	7	5
	Minor abnormal: minor hyperintense/minor hypointense	10	2	4	3
	Severely abnormal/fluid-like	0	0	0	1
Bony defect or bony overgrowth	No bony defect or overgrowth	10	5	7	4
	Bony defect: depth < thickness of adjacent cartilage or overgrowth <50% of adjacent cartilage	5	4	3	3
	Bony defect: depth ≥ thickness of adjacent cartilage or overgrowth ≥50% of adjacent cartilage	0		1	2
Subchondral changes	No subchondral changes	20	8	8	4
	Minor edema-like marrow signal: maximum diameter <50% of the repair tissue diameter	15	1	3	3
	Severe edema-like marrow signal: maximum diameter ≥50% of the repair tissue diameter	10	0	0	2
	Subchondral cysts ≥5 mm or osteonecrosis-like signal	0	0	0	0
Total score			96.1	80.2	71.7

## Data Availability

Data are contained within the main text of this article.
